# Gender differences in Greek centenarians. A cross-sectional nation-wide study, examining multiple socio-demographic and personality factors and health locus of control

**DOI:** 10.1186/1471-2318-11-87

**Published:** 2011-12-21

**Authors:** Xanthi Tigani, Artemios K Artemiadis, Evangelos C Alexopoulos, George P Chrousos, Christina Darviri

**Affiliations:** 1 First Department of Pediatrics, Children's Hospital Aghia Sofia, School of Medicine, University of Athens, Thivon & Papadiamantopoulou Str., GR-115-27, Athens, Greece; 2 Postgraduate Course Stress Management and Health Promotion, School of Medicine, University of Athens, Soranou Ephessiou Str., 4, GR-115-27, Athens, Greece

**Keywords:** centenarians, Greece, gender, longevity, personality

## Abstract

**Background:**

Centenarians are exceptional ageing paradigms, offering valuable information on achieving longevity. Although, there are several studies examining different biomedical factors as determinants of longevity in centenarians, little is known about gender differences with respect to personality traits and health locus of control.

**Methods:**

Nation -wide study carried out in Greece, between 2007 and 2010. Our final sample of analysis consisted of 400 centenarians who reported on sociodemographic, disease-related and personality factors and health locus of control (HLC). Gender differences were investigated by simple nonparametric comparisons. Bivariate correlations between personality factors and internal and external HLC were obtained.

**Results:**

Women centenarians outnumbered men by a ratio of 1.68 to 1. Significant gender sociodemographic differences were noted, with men reporting less often widowhood, more often centenarian 1st degree relatives and smoking. Higher BMI score was measured in males than females. Concerning personality variables, females were more reward-dependent and adaptable than men, while men were more optimistic than women. No differences were found on health locus of control profile between the genders. Positive correlations between self-directness and spirituality with internal locus of control in men and negative correlations between optimism and external locus of control in women emerged as the main gender disparities in the correlation analyses. Self-directness in men and optimism in women were consistently correlated with the two HLC subscales.

**Conclusions:**

Gender differences should be incorporated in future basic research and epidemiological studies of longevity. Informed policies on ageing and wellbeing programs should also take into account gender issues to increase efficacy by targeting health locus of control.

## Background

Centenarians have attracted much research interest, as paradigms of exceptional longevity and healthy ageing, as having succeeded to evade major chronic diseases and enjoy long autonomous lives [1]. The gradual increase of both the number of centenarians and the life expectancy in developed countries, has stimulated investigators to search for putative determinants of longevity that would be taken into account when designing and applying new public health policies [2,3].

So far, numerous factors, both genetic and environmental have been reported to influence longevity and special emphasis has been given to dietary, physical activity and psychosocial factors [4-6]. When compared to younger age groups, centenarians, in general, follow a healthier lifestyle along their life span, are more resilient to stress and adversity and they tend to have more positive thoughts about their self and life [7-13].

As far as personality traits are concerned, and despite high variability, these converge to frequent general characterizations of a "robust" but relaxed personality, extroversion, openness, efficiency and low neurotism [for a detailed review see [14]]. In centenarians, an apprehensive personality type and lack of self-efficiency and optimism have been correlated with poorer subjective health and happiness, respectively [15,16].

However, as outlined by Poon et al., psychosocial studies on longevity are less robust than biomedical ones, whereas personality measures collected are restricted to specific traits that do not provide a holistic picture of the centenarians' personality profile [14].

Health locus of control (HLC) represents the degree of personal responsibility relative to health outcomes. It is categorized into "external" and "internal", regarding the extent to which external (e.g. powerful others, chance etc.) or internal (e.g. the "self") factors influence health [17]. HLC has been associated with quality of life, subjective sense of well-being, psychological well-being and health-related behaviors such as diet and smoking [18-23]. As such, HLC seems to be a key element of general health and well-being that deserves to be investigated in different population settings. To our knowledge, HLC and its putative correlations with personality characteristics has never been investigated in centenarians.

In Greece, there are few studies that examined factors such as sociodemographic, genetic, disease-related and simple qualitative reports of centenarians [8,24-28]. The largest reported samples came from a Stathakos et al. study (N = 489), that showed that a high proportion of centenarians were in "optimal" condition, meaning relatively healthy, autonomous and socially active [27]. Gender differences among several sociodemographic and disease-related variables were also reported in the same study.

Our first study aim was to identify gender differences relative to sociodemographic and disease-related variables in Greek centenarians. Secondly, in the same group of centenarians, we examined for the first time to our knowledge, gender psychosocial profile that include personality, character and temperament profiles and health locus of control.

## Methods

### Sample

This was a cross-sectional study of centenarians conducted in Greece, during the period January 2007 to January 2010. For sampling purposes, a letter was sent to official services that hold catalogues of registered centenarians, such as the municipalities. In addition, a letter describing the study purposes was sent to both rural and urban health centers and hospitals, in order to increase the chances of locating as many centenarians as possible. Through a network of health professionals, 508 centenarians were identified and approached, from different geographic regions of the country, covering ten out of the eleven geographic divisions of Greece. The only exception was the division of East Macedonia and Thrace, due to lack of local resources. Exclusion criteria were severe cognitive dysfunction, meaning diagnosed type of dementia (e.g. Alzheimer) or use of dementia drugs according to prescription health books and severe hearing problems. The final sample consisted of 400 centenarians, as 23 people (4.5%) were excluded from the analysis and 85 centenarians (16.7%) refused to participate.

For age validation purposes, all respondents provided their official identification νcard, a mandatory document for all Greek citizens, on which the birth date is indicated. Trained healthcare personnel carried out the interviews, which were usually conducted in two consecutive visits in order to avoid distressing and tiring the respondents. Interviews were conducted in the subjects' residences (including nursing homes). No proxies were allowed.

### Measurements

Interviews were structured around four principal measurements covering (a) sociodemographic variables, (b) disease-related variables, (c) health locus of control and (d) personality variables.

a) Sociodemographic variables: Age, habitat status (living alone, living with family, living in nursing houses), type of habitat (rural or urban regions), family status, number of children, number of centenarian first degree-relatives, education, income and smoking (yes, no, occasionally). Family status (married, single, divorced or widowed) was limited to two categories widow (-er) and non-widow (-er), as the number of single and divorced centenarians was extremely low. Education included seven categories (illiterate, attending some classes of primary school, graduating primary school, attending some classes in secondary school, graduating from secondary school, attending some university classes, graduating from university). These seven categories of years of education were merged into to two broader basic categories, namely illiterate, and literate as descriptive analysis showed that most centenarians were illiterate and only a small proportion of them had high school or university education. The illiterate category included centenarians who have received no formal education at all, or have not completed primary school. All other responses regarding years of education were included in the literate category. Finally, monthly income was divided in two major categories, using the lowest pension given in Greece as a cut-point (≥500 Euros).

b) Centenarians reported on their current major disease(s) as diagnosed by a physician, using a long list of disease states. For this study, only general disease categories were used, except for diabetes mellitus which was separately asked. The decision to set diabetes mellitus as a separate, common category was based on the fact that it has distinct phenotypical and prognostic characteristics from the other three categories, thus it represents a syndrome deserving special attention. The general disease categories were: cardiovascular, neoplastic and neuropsychiatric diseases. For each individual, height and weight was measured at the site of first encounter. All weighing machines were of the same brand name and model. Body mass index (BMI) was calculated as (Kg/m2), according to World Health Organization recommendations. We classified for both men and women of the study into three groups: normal (< 25 kg/m2), overweight (25-29.9 kg/m2) and obese (≥30 kg/m2).

c) *Health Locus of Control (HLC)*. HLC questionnaire was administered in order to examine the extent to which centenarians believed that they had control over their health (internal locus of control), or this was primarily due to fate, chance, or other peoples' influence (external locus of control) [17]. This is an eleven item Likert-type scale consisting of six external and five internal items scoring from one (strongly disagree) to six (strongly agree). Individual internal and external HLC scores were calculated by summing up the items. The Cronbach's alpha total score was moderately satisfactory, 0.694 (for internal HLC 0.770 and 0.671 for external HLC).

d) Personality variables: *Temperament and Character Inventory (TCI)*. The Cloninger's TCI (9th version) has been designed to examine differences in seven major dimensions of temperament and character. Subjects have to respond to statements as false (= 0) or true (= 1) [29]. The final score for each subscale was produced by summing up item scores after appropriate reversing of negatively scored items. According to Cloninger, temperament refers to the automatic emotional responses that individuals have towards experiences, is stable throughout life and heritable. On the contrary, character is formulated throughout life influenced by socio-cultural and other environmental factors, and involves all those values, beliefs, concepts and goals that influence the meaning people give to experiences [29]. Linguistic validated process comprised of two independent forward translations into Greek and one backward performed by a native English speaker.

Using TCI, we explored two temperament dimensions. The first one is *harm avoidance*, a thirty-five item scale. Scoring high in harm avoidance describes a person inhibited and shy, careful, apprehensive, nervous and rather pessimistic, even in situations that most people would not worry. On the contrary, a low score characterizes a rather relaxing, courageous daring and optimistic person, confident in most societal conditions. *Reward dependence *is a twenty-four items scale, when scoring high, people tend to be sensitive, kind, more dependent and easy to socialize.

They empathize with the feelings of other people, but sometimes their objectivity can be influence by thirds. On the other hand, low scoring better describes people that are tough-minded, detached, practical and not socially sensitive. They can be very independent from sentimental situations and have more self-centered interpretation of social clues.

Character traits included self-directness and self-transcendence. *Self-directness *consists of forty-four items, with people scoring high described as mature, responsible, self-sufficient, with high self-esteem and effective into pursuing their main goal. On the other hand, low self-directed people are immature, irresponsible, fragile, less able to pursuit goals and more susceptible to external pressures. *Self-transcendence *includes thirty-three items, with high scorers in this trait usually being spiritual, creative, selfless, modest and able to accept both success and failure, but sometimes also seen as naive. On the contrary, low scorers tend to be more impatient, materialistic and, not enjoying uncertainty; as they grow they tend to have greater difficulty into accepting death and suffering, but admired for their scientific objectivity. Reliability analysis for total TCI was high (Cronbach's alpha 0,717) and similarly for each TCI trait Cronbach's alphas were the following: 0.769 (Harm Avoidance), 0.844 (Reward Dependence), 0.840 (Self-directness), 0.783 (Self-transcendence).

*International Personality Item Pool (IPIP) scales*. IPIP was used to measure adaptability, spirituality, and optimism [30,31]. Participants were asked to rate their agreement with several statements based on a 5-point Likert-type scale (1 = strongly agree, 5 = strongly disagree). The total score for each scale was produced by summing up items. High scores reflect higher individual accordance with each subscale attributes.

*Adaptability *is an 8-item scale that explores the ability of people to adjust and fit to different circumstances. *Spirituality *is a 9-item scale used to explore individual spiritual beliefs. Finally, *optimism *is a 10-item scale exploring individual expectations for the future. Reliability Cronbach's alpha tests for adaptability spirituality and optimism were high, 0.749, 0.770 and 0.844 respectively.

## Statistical Analyses

Statistical calculations were performed using SPSS for Windows (version 18.0.3) statistical software (SPSS Inc., Chicago, IL). Chi-square tests were used for comparing percentages between genders. Non-parametric numeric comparisons (e.g. Mann-Whitney test) were used after testing for normality with Q-Q plots and Kolmogorov-Smirnov test (if p < 0.05, the null hypothesis of normality is violated). Due to our large sample, means, ranges and standard deviations are presented to allow proper comparisons with other studies. Bivariate correlations between personality variables and HLC were made using Pearson rho coefficient. For each analysis cases with missing values were excluded. A significance level of 0.05 was used for all tests.

## Results

### Sociodemographic and disease-related characteristics of centenarians by gender

Women (N = 251) outnumbered men (N = 149) by a ratio of 1.68 to 1. Missing values were few (up to 2.7% at most), except for BMI, with missing values of 14.8% and 17.6% for males and females respectively, as certain participants refused to be measured. Scores of individual subscales were calculated only in the case of no missing items, otherwise they were declared as missing values. Sociodemographic characteristics analyzed by gender are presented in detail in Table 1. The mean age of the total sample was 101.85 years old, ranging from 100 to 109. No statistical differences were found in terms of mean age per gender group. The majority of centenarians were living in rural homes with at least one family member, while 8.7 and 7.9% of, males and females, respectively, were living in nursing homes.

**Table 1:** Sociodemographic and disease-related characteristics by gender group^1^

	Males (N = 149) (%)	Females (N = 251) (%)	p value
**Mean age in years old (SD) Range**	101.62 (1.88) 100-107	101.99 (2.13) 100-109	0.1

**Habitat status**			0.632

Home alone	38 (25.9)	53 (21.6)	

Home not alone	96 (64.4)	172 (68.5)	

Nursing home	13 (8.7)	20 (7.9)	

**Type of habitat**			0.134

Urban	33 (22.8)	74 (30.3)	

Rural	112 (77.2)	170 (69.7)	

**Family status**			< 0.0005*

Non widow(-er)	30 (20.1)	15 (5.9)	

Widow(-er)	119(79.9)	236(94.1)	

**Mean number of children (range)**	3.8 (0-12)	4.2 (0-11)	0.233

**Number of centenarian 1^st ^degree siblings**			0.023*

No	132 (88.6)	238 (94.8)	

One	12 (8.1)	12 (4.8)	

More than one	5 (3.6)	1 (0.4)	

**Education**			< 0.0005*

^2^Illiterate	32 (21.8)	139(55.6)	

^3^Literate	115(78.2)	111 (44.4)	

**Income**			0.21

< 500 euros	75 (51.7)	139 (58.9)	

≥500	70 (48.3)	97 (41.1)	

**Smoking**			< 0.0005*

Yes	21 (14.4)	2 (0.8)	

No	116 (79.5)	242 (97.2)	

Occasionally	9 (6.2)	5 (2)	

**BMI**			0.001*

Normal	75 (57.3)	119 (51.5)	

Overweight	47 (35.9)	58 (25.1)	

Obese	5 (3.8)	30 (12.9)	

**Diseases**			

Cardiovascular	36 (25.9)	59 (26.7)	0.965

Diabetes Mellitus	11 (7.9)	14 (6.3)	0.718

Cancer	28 (19.7)	66 (28)	0.094

Neuropsychiatric	2 (1.4)	11 (5)	0.141

SD: Standard deviation, BMI: Body Mass Index

^1^Categorical by categorical comparisons were made using chi-square tests. Gender differences in scale variables were assessed by non-parametric Mann-Whitney tests.

^2^Illiterate: Centenarians who have not received formal education at all or have not completed primary school.

^3^Literate: Centenarians who have graduated at least from primary school.

*p < 0.05 (two-tailed)

With regards to family status, most of centenarians were widowed, with significant prevalence of female widows. Mean number of children did not differ among gender groups. Interestingly, males reported having more often one or more centenarian first degree relative than females (p = 0.023). Illiteracy was greater among female centenarians, whereas the majority of males were literate (p < 0.005). Findings on centenarians' monthly income did not show any significant gender differences; with a great proportion of both genders receiving a pension lower than 500 euros. Regular and occasional smokers as well as overweight people were significantly more among male than female centenarians (p < 0.0005). No gender differences were found for major disease categories.

### Gender differences on personality characteristics and health locus of control among centenarians

The gender analysis of psychosocial variables, as shown in Table 2, revealed no significant differences in the two temperament subscales, namely self-directness and self-transcendence, with both gender groups reporting almost identical scores. On the contrary, a profound difference was found in the character subscale of reward dependence, with females scoring higher (mean score = 15.41, p = 0.009). For harm avoidance no difference was found between the gender groups.

**Table 2:** Psychosocial variables by gender group^1^

	Males (N = 149) (%)	Females (251) (%)	p value
**TCI subscales**			

Self-directness (SD)	29.4 (7.59)	29.14 (7.02)	0.67
Range	13-42	14-41	

Self-transcendence (SD)	16.73 (6.32)	16.75 (5.61)	0.66
Range	4-38	4-31	

Harm avoidance (SD)	14.71 (6.82)	15.67 (6.58)	0.23
Range	2-31	2-32	

Reward dependence (SD)	14.2 (4.26)	15.41 (4.3)	0.009*
Range	2-22	3-23	

**HLC subscales**			

Internal HLC (SD)	21.36 (4.85)	21.19 (4.29)	0.598
Range	6-30	5-30	

External HLC (SD)	21.6 (5.43)	21.81 (5.06)	0.772
Range	9-36	6-35	

**IPP subscales**			

Adaptability (SD)	26.58 (6.03)	27.92 (5.77)	0.027*
Range	11-40	10-41	

Spirituality (SD)	36.83 (6.54)	38.25 (5.45)	0.059
Range	12-45	17-45	

Optimism (SD)	38.02 (7.45)	36.15 (7.6)	0.011*
Range	16-50	14-50	

SD: Standard deviation, TCI: Temperament and Character Inventory, HLC: Health Locus of Control, IPP: International Personality Item Pool

^1 ^Gender differences in scale variables were assessed by non-parametric Mann-Whitney tests.

*p < 0.05 (two-tailed)

Health locus of control measured by two subscales, internal HLC and external HLC, revealed no statistical differences in terms of gender.

When IPP subscales were analyzed, significant gender differences were found for two out of the three different subscales. Female centenarians had higher mean scores in adaptability than men (p = 0.027). On the other hand, male centenarians had higher mean scores in optimism (p = 0.011). No gender differences were found in spirituality.

### Bivariate correlation analysis (Table 3)

Both men and women centenarians showed negative correlations between selfdirectness and external HLC and positive correlations between harm-avoidance and external HLC. Concerning internal HLC, positive correlations were noted with optimism for both genders. Men centenarians were distinguished from women by, showing a positive correlation between internal HLC and both self-directness and spirituality. Finally, only women centenarians who had higher optimism scores had lower external HLC scores. Thus, consistent and strong inverse correlations between one personality factor and the two subscales of HLC, were noted for self-directness (Internal HLC: r = 0.209, External HLC: r = -0.218) in men and for optimism in women (Internal HLC: r = 0.279, External HLC: r = -0.234).

**Table 3:** Bivariate correlations (Pearson rho) between TCI, IPP subscales and HLC subscales

	Males		Females	
	
	Internal HLC	External HLC	Internal HLC	External HLC
**TCI subscales**				

*Self-directness*	.209*	-.218*	.116	-.206**

*Self- transcendence*	.109	-.009	.098	.043

*Harm- avoidance*	-.058	.192*	-.127	.155*

*Reward-* *dependence*	.062	-.007	-.036	-.032

**IPP subscales**				

*Adaptability*	.101	-.007	.037	.043

*Spirituality*	.214**	.072	.105	.073

*Optimism*	.172*	-.025	.279**	-.234**

TCI: Temperament and Character Inventory, HLC: Health Locus of Control, IPP: International Personality Item Pool

** Correlation is significant at the 0.01 level (two-tailed)

* Correlation is significant at the 0.05 level (two-tailed)

## Discussion

In this study we examined gender differences of sociodemographic, disease-related, personality and health locus of control variables among Greek centenarians. Among the sociodemographic differences found, many confirmed previous studies (see below). Regarding personality traits, females were more reward-dependent and adaptable than men, while men were more optimistic than women. No gender differences were found on health locus of control. The more consistent and strong correlations of HLC were found for self-directness in men and optimism in women, suggesting that these two personality factors are key determinants of the perceived individual health responsibility.

Analyses of our data resulted in several basic gender discrepancies that partly confirmed other Greek and foreign studies. In particular, female predominance was lower than the previous Greek large scale study and other studies across the world [27,32-35]. Additional congruencies were noted regarding most centenarians living with others in rural homes, females being windowed more often than men and less educated and males having a greater BMI and smoking more often [27]. One discrepancy with the Stathakos et al. study was that in our study there were four times more centenarians living in nursing homes [27]. It is noteworthy, that the mean number of off-spring was similar for men and women (3.8 for males and 4.2 for females), but high with respect of the modern family structure in Greece, where the fertility ratio is currently 1.2. Most probably, this should be due to known trends in family programming in the Western world during the past century.

Interestingly, male centenarians reported more often having at least one centenarian first degree relative than females, implying a higher genetic predisposition in the longevity of men. This is supported by previous genetic and epidemiological studies [32]. Finally, there were no differences regarding major disease categories. Comparison with the previous Greek study is difficult due to different disease categorization; however, the earlier results off no gender difference concerning diabetes mellitus and cancer were confirmed [27].

Several differences of personality indices of longevity between male and females centenarians were found. To our knowledge, there is no other study addressing this issue. In our study, in general, women centenarians were more adaptable and sensitive, kind and socially capable (high reward dependence) than men, while men scored higher in optimism than women. Our results give further support to personality specific dominant features in men and women. High reward dependence and adaptability in women could be related to higher social sensitivity leading to more intimate and supportive relationships and greater chance of successful adaptation to different situations and, thus, less distress over the lifespan [29]. Men's high scores on optimism could reflect higher confidence and vigorous optimistic efforts, offering them a certain advantage in terms of adaptability to diversity and uncertainty [29].

These findings could also correspond to past contextual gender differences in Greece, when women were more family-oriented and restricted to a rather supportive role [36]. Moreover, men had to adapt to multiple adverse social conditions (such as wars and conflicts), which presumably could increase their resilience to stress and their self-efficacy, leading to higher optimism concerning future outcomes.

In general, significant correlations were noted between HLC and self-directness, harm-avoidance, spirituality and optimism. Men showed both strong positive and negative correlations between self-directness and internal and external HLC, respectively. Self-directness has been linked with greater self-acceptance promoting greater self-responsibility and also self-efficacy to specified individual goals [17,29].

On the other hand, optimism was consistently correlated with HLC among women, indicating that more optimistic women have a more internalized HLC. Optimism and a positive mood state have been indirectly linked to an internalized HLC, by fostering less risky health-related behaviors [37].

For the first time, higher spirituality in male centenarians was strongly correlated with high internal HLC. To date, there is no proven relation between spirituality and HLC, although theoretically, higher spirituality would lead to a more internalized health locus of control [38]. Interestingly, high spirituality was linked with longevity previously [39-41]. Finally, not surprisingly, centenarians' harm-avoidant temperament was positively correlated with higher external HLC, suggesting that in both genders, inclination to inhibition, uncertainty and worry enhance health dependence to external resources such as physicians etc. [17,29].

This study has some limitations that need to be addressed. First of all, the cross sectional design does not allow etiological interpretations. Future cross-sectional and follow-up studies should focus on gender differences across various age groups that would give a more robust insight into gender disparities through extreme longevity. Secondly, there is always the chance of information or recall bias, particularly for psychosocial variables. We should take into account that centenarians are often very proud of themselves, especially when they are asked to be studied as exceptional paradigms, so reality, and especially past reality, may be distorted to a direction of "how I should have been" and not "how I was actually". The solution of confirmation by proxy, as some researchers suggested in the past, entails the risk of precipitated distortion, because past interpersonal relationships can easily intrude the proxy's image of the centenarian. Also, a limited bias or misclassification might be introduced by the relative low Cronbach's alpha for external HLC and by the absence of available standardized cut-off points for HLC. Thirdly, many others psychosocial factors have to be taken into account in order to develop a comprehensive theoretical model on centenarians "healthy" profile. Finally, excluding centenarians of severe cognitive and hearing problems may increase the probability of obtaining a relatively selective sample of "healthy" centenarians. However, we should note that this is a large sample, meaning 25% of all centenarians registered in the 2001 census with a country-wide distribution.

## Conclusions

In conclusion, this group of centenarians is important for research on successful ageing, taking into account gender differences on both the demographical, health and personality level. According to our preliminary findings, self-directness for men and optimism for women emerged as significant putative factors of health locus of control, a valuable public health measure. Future research on the psychosocial profile of centenarians should adopt a more comprehensive and modeling approach by gender group. This gender informed approach could increase the efficiency of future ageing and well-being policies and programs.

## Competing interests

The authors declare that they have no competing interests.

## Authors' contributions

CD had the conception of this study. CD and XT designed the study. XT and AKA wrote the paper. Analysis and interpretation of the data was done equally by XT, AKA, ECA and CD. Revision and final approval were made by ECA, CD and GPC. All authors have read and approved the final manuscript.

## Pre-publication history

The pre-publication history for this paper can be accessed here:

http://www.biomedcentral.com/1471-2318/11/87/prepub
